# The genus *Eurymeros* Bhat (Hymenoptera, Braconidae, Alysiinae) newly recorded from China

**DOI:** 10.3897/BDJ.11.e100784

**Published:** 2023-08-08

**Authors:** Jia-Chen Zhu, Shu-Qian Fang, Qing-Yan Zhao, Jun-Li Yao, Yan-Qiong Peng, Van Achterberg Cees, Xue-Xin Chen

**Affiliations:** 1 State Key Laboratory of Rice Biology and Ministry of Agriculture and Rural Affairs Key Lab of Molecular Biology of Crop Pathogens and Insect Pests, Institute of Insect Sciences, Zhejiang University, Hangzhou 310058, China, Hangzhou, China State Key Laboratory of Rice Biology and Ministry of Agriculture and Rural Affairs Key Lab of Molecular Biology of Crop Pathogens and Insect Pests, Institute of Insect Sciences, Zhejiang University, Hangzhou 310058, China Hangzhou China; 2 Beneficial Insects Institute, Fujian Agriculture & Forestry University, Fuzhou, China Beneficial Insects Institute, Fujian Agriculture & Forestry University Fuzhou China; 3 Key Laboratory of Tropical Forest Ecology, Xishuangbanna Tropical Botanical Garden, Chinese Academy of Sciences, Kunming, China, Kunming, China Key Laboratory of Tropical Forest Ecology, Xishuangbanna Tropical Botanical Garden, Chinese Academy of Sciences, Kunming, China Kunming China

**Keywords:** Hymenoptera, Braconidae, Alysiinae, Alysiini, new record, Oriental Region, China

## Abstract

**Background:**

Alysiinae Leach is a species-rich subfamily in Braconidae, of which several species play an important role in biological control. The monotypic genus *Eurymerostumespiraculum* Bhat, 1980 was discovered in Tibet and Yunnan provinces for the first time, representing the first record of the genus *Eurymeros* Bhat, 1980 (Braconidae, Alysiinae) in China.

**New information:**

The rare genus *Eurymeros* Bhat, 1980 (Braconidae, Alysiinae) and its only known species, *E.tumespiraculum* Bhat, 1980, are newly recorded from China. The morphological variation of the Chinese specimens is described and illustrated.

## Introduction

The subfamily Alysiinae (Hymenoptera, Braconidae) is a quite large subfamily in Braconidae, containing over 2450 species worldwide (Chen and van Achterberg 2019). It is characterised by the outwardly directed ('exodont') mandibles which do not meet when they are closed (Shaw and Huddleston 1991, van Achterberg 1993, Belokobylskij and Kostromina 2011). There are two tribes Alysiini and Dacnusini, in this subfamily, with the first tribe possessing nearly twice the number of genera (71 genera) compared with the Dacnusini (33 genera) (Yu et al. 2016, Zhu et al. 2017). Despite the monophyly of Alysiinae being widely accepted, whether the tribe Alysiini is a monophyletic group is still controversial. Recent research on the phylogeny of Braconidae showed that four species and genera of Alysiini formed a clade with the Dacnusini (Jasso-Martínez et al. 2022).

The *Eurymeros* Bhat, 1980 is a small and rare Oriental genus from Alysiini and, so far, was only known from India (Gupta and van Achterberg 2022). This genus can be recognised by the extremely widened dentate hind femur. The biology of *Eurymeros* is unknown, but all members of the subfamily Alysiinae are koinobiont endoparasitoids of larval cyclorrhaphous Diptera (Wharton 1984, van Achterberg 1993, Chen and van Achterberg 2019). Bhat (1980) established this genus and included only the type species *Eurymerostumespiraculum* Bhat, 1980. Later, Sharma (1983) published two new species from India: *E.gibbosa* Sharma, 1983 and *E.mangifera* Sharma, 1983, but, recently, these were transferred to the genus *Euscelinus* Westwood, 1882 of the subfamily Doryctinae (Gupta and van Achterberg 2022).

In this paper, we discovered the species *E.tumespiraculum* in China for the first time and described the variation of the newly-recorded specimens.

## Materials and methods

The examined specimens were collected by Malaise traps and glued on card points. They are deposited in the the Parasitic Hymenoptera Collection, Institute of Insect Sciences of the Zhejiang University (ZJUH) and in the insect collection of the Fujian Agricultural and Forestry University (FAFU).

For the recognition of the subfamily Alysiinae, see van Achterberg (1990) and van Achterberg (1993). For additional references, see Yu et al. (2016). The terminology and measurements used follow van Achterberg (1988) andvan Achterberg (1993). The following abbreviations are used: POL – postocellar line; OOL – ocular – ocellar line, measured from ocellus directly to eye; OD – maximum diameter of lateral ocellus; medial length of the first tergite is measured from the apex of adductor to the apex of tergite. Descriptions and measurements were made under a Leica M125 stereomicroscope. Photographs were made with a Keyence VHX-2000 digital microscope and the photos were slightly processed (mainly cropped and modification of background) in Photoshop CC.

## Taxon treatments

### Eurymeros tumespiraculum

Bhat, 1980

12E81628-ED79-50BC-BCEA-3BCDCFB8F8A2

#### Materials

- **Type status:** Other material. **Occurrence:** recordedBy: Xingzhou Ma; individualCount: 1; sex: female; lifeStage: adult; occurrenceID: 939AB025-296B-51B4-A86D-93E2E99F7317; **Taxon:** scientificName: Eurymerostumespiraculum; class: Insecta; order: Hymenopetra; family: Braconidae; genus: Eurymeros; specificEpithet: tumespiraculum; **Location:** country: China; stateProvince: Tibet; locality: PailongXiang, Polonggou; verbatimCoordinates: 30°1'13"N, 94°59'48"E; **Identification:** identifiedBy: Jiachen Zhu; dateIdentified: 2020; **Event:** samplingProtocol: malaise trap; startDayOfYear: 1/8/2019; endDayOfYear: 16/8/2019; **Record Level:** collectionCode: Insects; basisOfRecord: PreservedSpecimen
- **Type status:** Other material. **Occurrence:** recordedBy: Lang YI; individualCount: 1; sex: female; lifeStage: adult; occurrenceID: 28DB30D9-542B-5755-A080-6175E4EDBA0E; **Taxon:** scientificName: Eurymerostumespiraculum; class: Insecta; order: Hymenopetra; family: Braconidae; genus: Eurymeros; specificEpithet: tumespiraculum; **Location:** country: China; stateProvince: Yunnan; locality: Gaoligong Mountain; verbatimElevation: 1373 m; verbatimCoordinates: 25°18′36.08″N, 98°47′40.65″E; **Identification:** identifiedBy: Shuqiang Fang; dateIdentified: 2022; **Event:** samplingProtocol: malaise trap; startDayOfYear: 30/10/2019; endDayOfYear: 15/11/2021; **Record Level:** collectionCode: Insects; basisOfRecord: PreservedSpecimen
- **Type status:** Other material. **Occurrence:** recordedBy: Lang YI; individualCount: 1; sex: female; lifeStage: adult; occurrenceID: 96E037E0-D033-56FB-AC1F-F1A90764657A; **Taxon:** scientificName: Eurymerostumespiraculum; class: Insecta; order: Hymenopetra; family: Braconidae; genus: Eurymeros; specificEpithet: tumespiraculum; **Location:** country: China; stateProvince: Yunnan; locality: Gaoligong Mountain; verbatimElevation: 1373 m; verbatimCoordinates: 25°18′36.08″N, 98°47′40.65″E; **Identification:** identifiedBy: Shuqiang Fang; dateIdentified: 2022; **Event:** samplingProtocol: malaise trap; startDayOfYear: 15/11/2021; endDayOfYear: 30/11/2021; **Record Level:** collectionCode: Insects; basisOfRecord: PreservedSpecimen
- **Type status:** Other material. **Occurrence:** recordedBy: Lang YI; individualCount: 1; sex: female; lifeStage: adult; occurrenceID: 6082BB23-AA5A-5B77-916C-43154E4177CA; **Taxon:** scientificName: Eurymerostumespiraculum; class: Insecta; order: Hymenopetra; family: Braconidae; genus: Eurymeros; specificEpithet: tumespiraculum; **Location:** country: China; stateProvince: Yunnan; locality: Gaoligong Mountain; verbatimElevation: 1373 m; verbatimCoordinates: 25°18′36.08″N, 98°47′40.65″E; **Identification:** identifiedBy: Shuqiang Fang; dateIdentified: 2022; **Event:** samplingProtocol: malaise trap; startDayOfYear: 15/03/2020; endDayOfYear: 30/03/2020; **Record Level:** collectionCode: Insects; basisOfRecord: PreservedSpecimen
- **Type status:** Other material. **Occurrence:** recordedBy: Lang YI; individualCount: 2; sex: female; lifeStage: adult; occurrenceID: EA994528-6742-5240-AC6A-E8B2DDCE91E3; **Taxon:** scientificName: Eurymerostumespiraculum; class: Insecta; order: Hymenopetra; family: Braconidae; genus: Eurymeros; specificEpithet: tumespiraculum; **Location:** country: China; stateProvince: Yunnan; locality: Gaoligong Mountain; verbatimElevation: 1373 m; verbatimCoordinates: 25°18′36.08″N, 98°47′40.65″E; **Identification:** identifiedBy: Shuqiang Fang; dateIdentified: 2022; **Event:** samplingProtocol: malaise trap; startDayOfYear: 30/06/2020; endDayOfYear: 15/07/2020; **Record Level:** collectionCode: Insects; basisOfRecord: PreservedSpecimen

#### Description

Female, Length of body 3.8–4.3 mm (excluding ovipositor; Figs 1a, 3a), of fore-wing 3.0–4.1 mm.

**Head**. Transverse, width of head 1.8–2.2 times its lateral length in dorsal view (Figs 1d, 3d); antenna complete, one with 28 segments and the other one with 27 segments (Fig. 1a). The length of antenna 0.8 times as long as the body; third segment (including annellus) 1.5–1.6 times longer than fourth segment, length of third and fourth segments 3.4–4.0 and 1.9–2.3 times their width, respectively (Fig. 1b); length of maxillary palp 1.9 times the height of head (Fig. 3b); eye in dorsal view 1.8–2.25 times as long as temple (Figs 1d, 3d); eye in lateral view 1.4–1.5 times higher than wide (Figs 1c, 3c); frons largely smooth; vertex and temple smooth; OOL:diameter of ocellus: POL = 5–8:2.3–5:3; face 1–1.3 times wider than high, rather convex medially and coarsely punctate; clypeus wide, near rectangle, smooth and finely rugose apical, distinctly separated from face in colouration (Figs 1b, 3b); malar space absent; mandible with 3 teeth, dorsal tooth enlarged, lobe-shaped (Figs 1c, 4f), ventral tooth medium-sized and lobe-shaped, middle tooth small and slightly acute, with minute incision between first and second teeth, medial length of mandible 0.9–1.2 times its maximum width.

**Mesosoma**. Length of mesosoma 1.5–1.6 times its height in lateral view (Figs 1e, 3e); medio-anteriorly pronotum inconspicuously crenulate, remainder of pronotum smooth (Figs 1f, 3f); epicnemial area rugose; precoxal sulcus crenulate and groove gradually deeper, not reaching middle coxa; pleural sulcus narrowly crenulated ventrally; episternal scrobe round and deep; metapleuron largely smooth medially, but slightly crenulated ventrally (Figs 1e, 3e); mesoscutum smooth with median oval depression situated a little above the posterior margin in the apical half; notauli present anterior one fourth, shallow; scutellum smooth, scutellar sulcus deep, with one longitudinal carina, sulcus 3.3 times wider than its maximum length (Figs 1f, 3f); surface of propodeum distinctly rugose at middle, with a medio-longitudinal carina present at basal 0.3–0.5 of propodeum, areola present posteriorly, but with coarsely rugose-reticulation (Fig. 4a).

**Wings** (Figs 2a, 4b). Pterostigma wide, 5.0–­5.1 times longer than its maximum width. Vein r issuing from middle of pterostigma. r:3-SR:SR1 = 4:16–19:28–39; 1-SR+M not or slightly sinuate; SR1 curved; r 0.6 times as long as width of pterostigma; 2-SR of fore-wing slightly antefurcal or distinctly antefurcal; 1-CU1:2-CU1 = 1: 6–6.5; 3-CU1 distinctly longer than CU1b; 2-SR:3-SR:r-m = 13–15:20–22:7; m-cu postfurcal, converging to 1-M posteriorly; first subdiscal cell 4.1–4.3 times as long as wide; M+CU1 largely unsclerotised.

**Legs**: Hind coxa rather smooth (Fig. 1e) or densely punctate (Fig. 4e), without ventro-basal tubercle, rather elongated, longer than fore and middle coxa. Hind femur distinctly widened, coarsely punctate and with long setae, with nine teeth (one large, three medium-sized, four small and one combined tooth with one medium tooth and one small tooth apically) laterally, seven medium teeth inner laterally (Figs 2c, 4e); hind basitarsus 0.34 times as long as hind tibia. Ratio of hind femur:hind tibia:hind tarsus:hind basitarsus = 1.22:1.61:1.19:0.50.

**Metasoma**: Metasoma elongated (Figs 2b, 4d); first tergite smooth, apical two fifths with irregular longitudinal carinae, remaining tergites smooth; first tergite widened from base to apex, apical width 2.1–2.4 times longer than its basal width (Figs 2b, 4d); dorsope large and distinct; total visible length of ovipositor sheath 0.9–1.2 mm and 0.8–1 times as long as hind femur (Figs 2b, 4c).

**Colour**. Blackish-brown (Figs 1a, 3a); mandible, posterior area of clypeus, palpi, pronotum, fore and middle legs, trochanter and trochantellus of hind legs, apical third areas of hind femur yellowish-brown; pterostigma and anterior part of veins brown, remainder of veins and wing membrane hyaline. Head black; eyes grey; whole clypeus (Fig. 1b) or only its posterior area (Fig. 3b) yellowish-brown; mandibles yellowish with dark brown margins, median tooth dark brown; palpi off white; ocelli transparent; antenna with scape and pedicel yellowish-brown and flagellum brown; mesoscutum yellowish-brown, except median depression and area around median depression brown or entirely brown (Figs 1f, 3f); scutellum and propodeum black; legs in general pale yellow to brown gradually with fore- and mid-leg coxae and trochanters yellowish-white, fore- and mid-femora and tibiae yellowish-brown, tarsi light brown, hind coxa light brown laterally to dark brown (Fig. 4e) and apical three fourths of hind femur black, basal one fourth yellowish-white and extreme basal margin brown; hind tibia gradually darker from apical one fifth pale brown to two fifths apically brown and remaining light brown, hind tarsus brown; wings hyaline with pterostigma dark brown; first tergite of metasoma black, following tergites reddish-brown dorsally and yellowish laterally; ovipositor brown.

**Variation**: Body length (including ovipositor) 3.8−4.5 mm; antennal segments of female 25−28; 1-SR+M 0.3−0.4 mm; hind femur distinctly widened with six to nine teeth (one large, three to four teeth medium, two to four teeth small and one combined by one medium tooth and one small tooth apically) laterally, 7 medium inner teeth laterally or 6 medium teeth and one combined tooth with one medium tooth and one small inner tooth apico-laterally; lateral length of first tergite 0.4−0.5 mm.

#### Distribution

**Figure 5.**
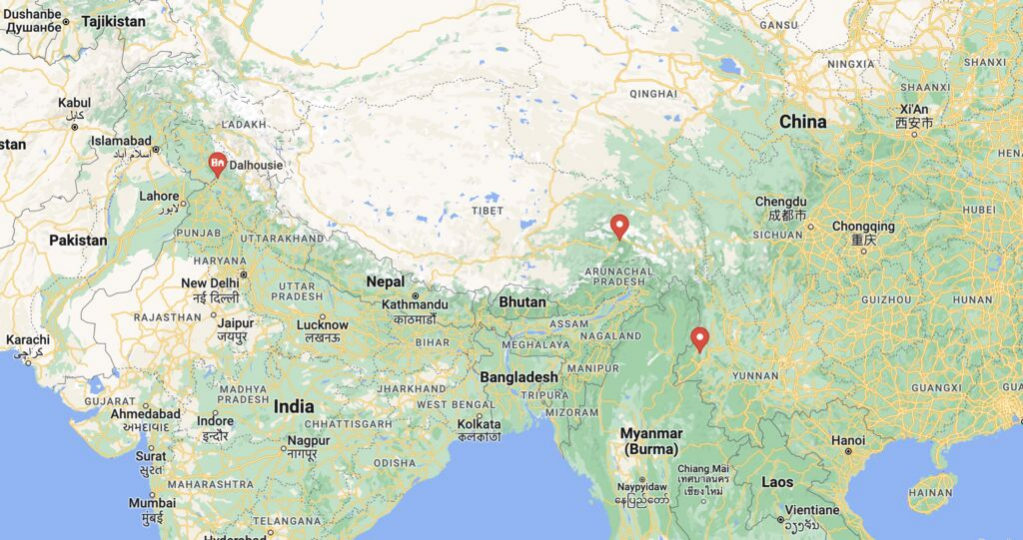
Collection localities of *Eurymerostumespiraculum* Bhat.

India: Himachal Pradesh: Dalhousie; China: Tibet, Yunnan. (Fig. 5)

#### Biology

unknown

## Supplementary Material

XML Treatment for Eurymeros tumespiraculum

## Figures and Tables

**Figure 1a.**
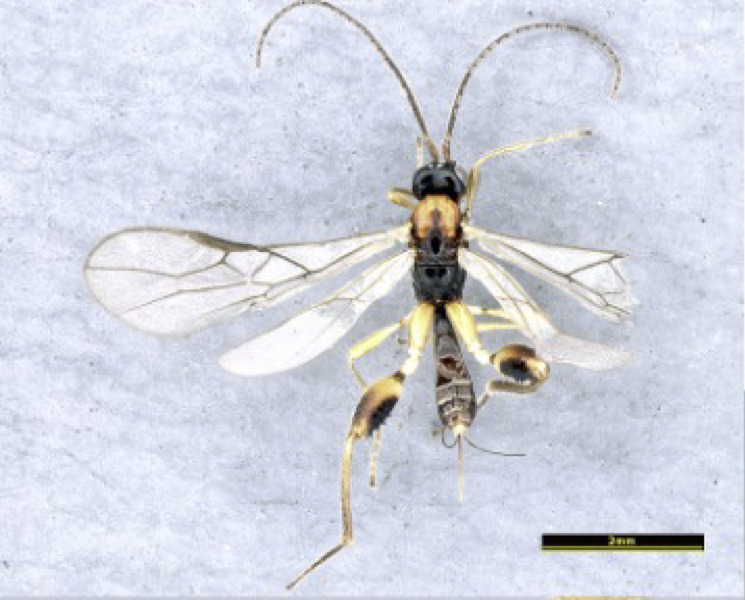
habitus, dorsal aspect;

**Figure 1b.**
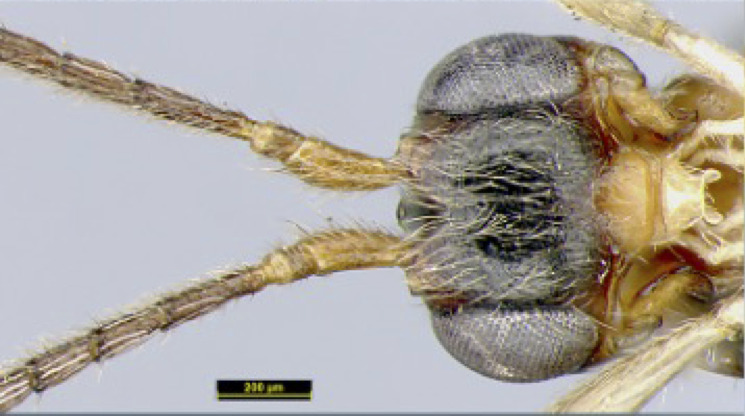
head, anterior aspect;

**Figure 1c.**
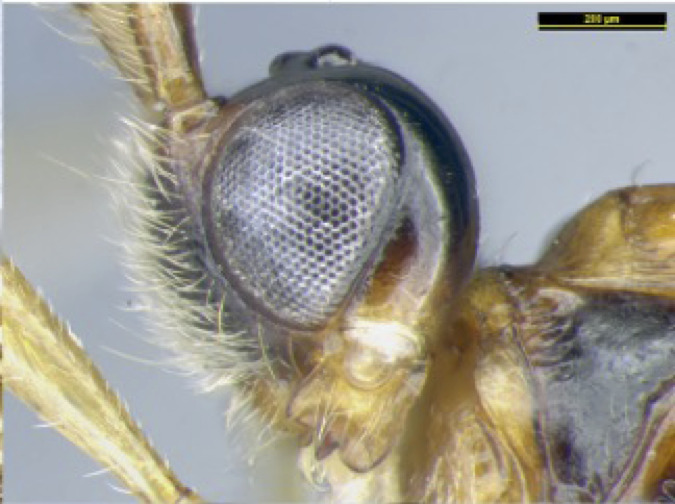
head, lateral aspect;

**Figure 1d.**
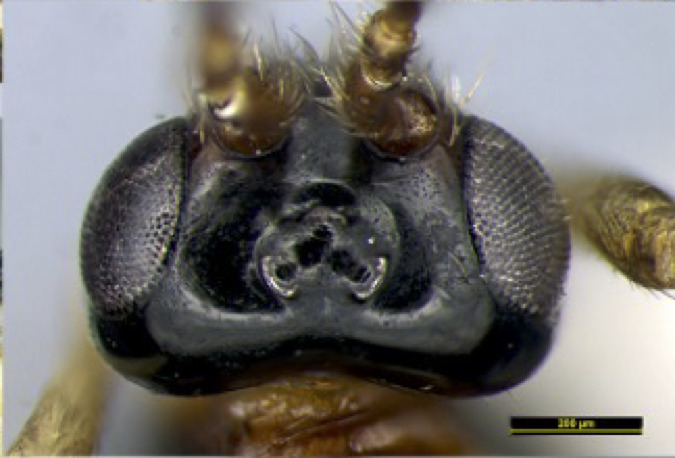
head, dorsal aspect;

**Figure 1e.**
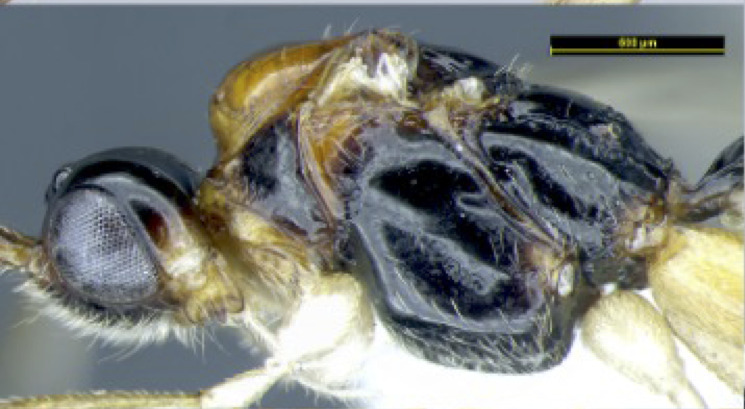
head and mesosoma, lateral aspect;

**Figure 1f.**
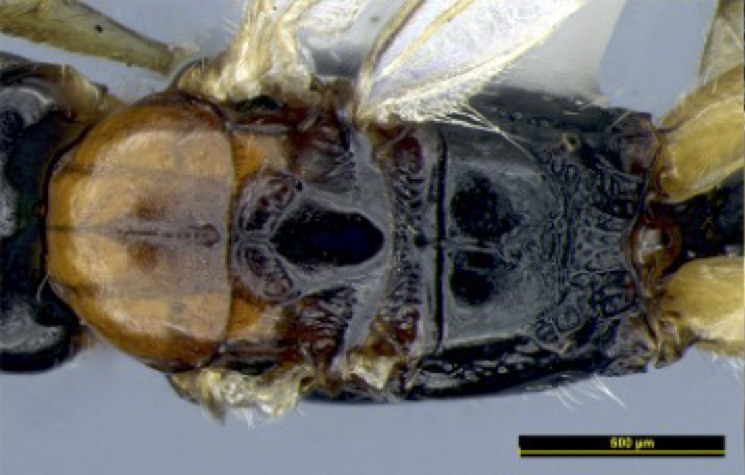
mesosoma, dorsal aspect.

**Figure 2a.**
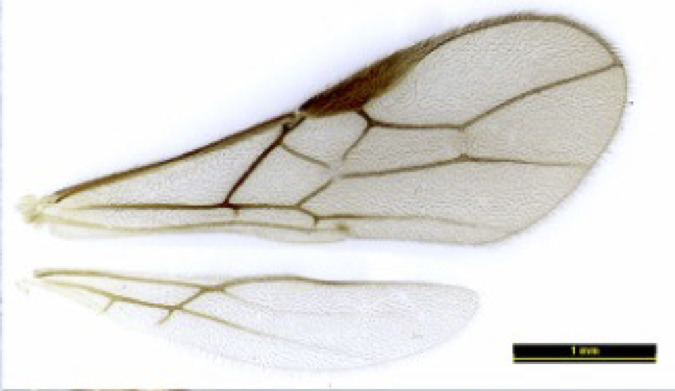
wings;

**Figure 2b.**
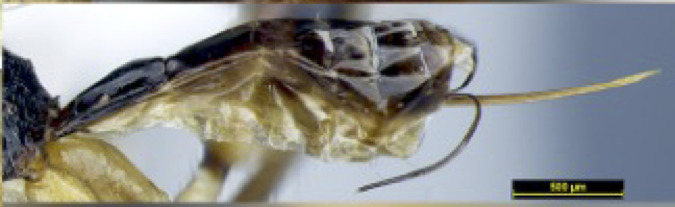
metasoma and ovipositor, lateral aspect;

**Figure 2c.**
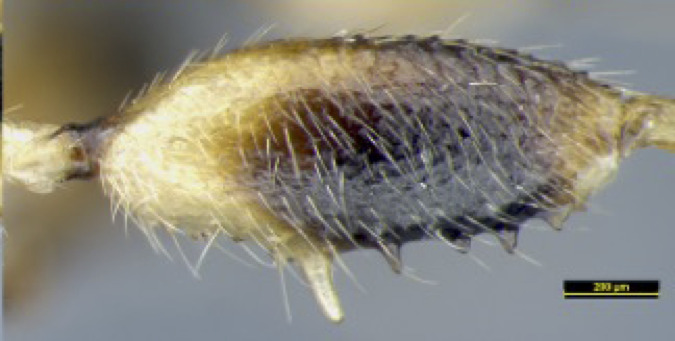
hind femur, lateral aspect.

**Figure 3a.**
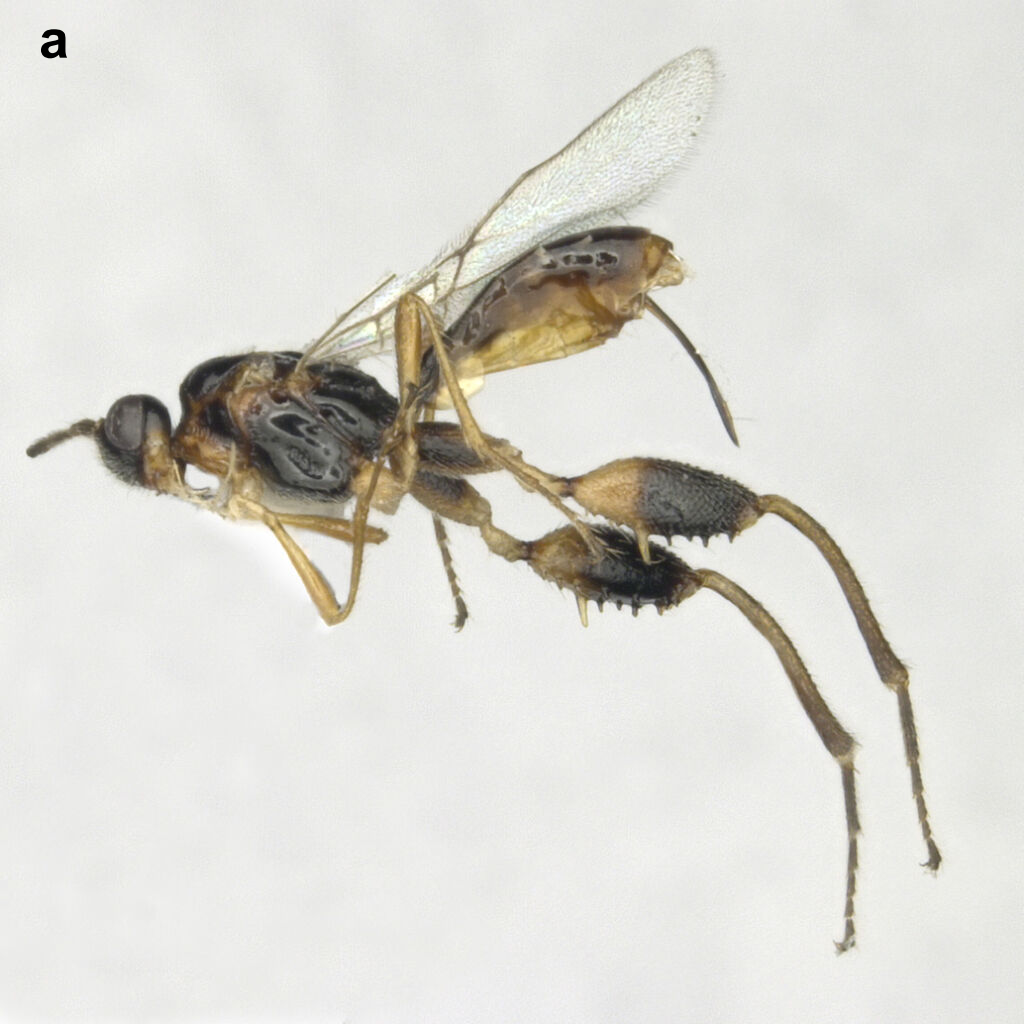
habitus, lateral aspect;

**Figure 3b.**
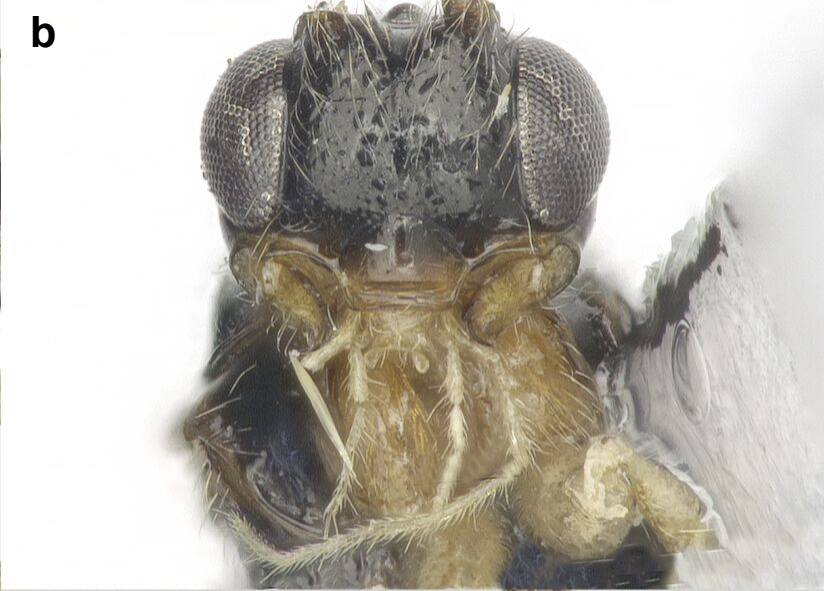
head and palpi, anterior aspect;

**Figure 3c.**
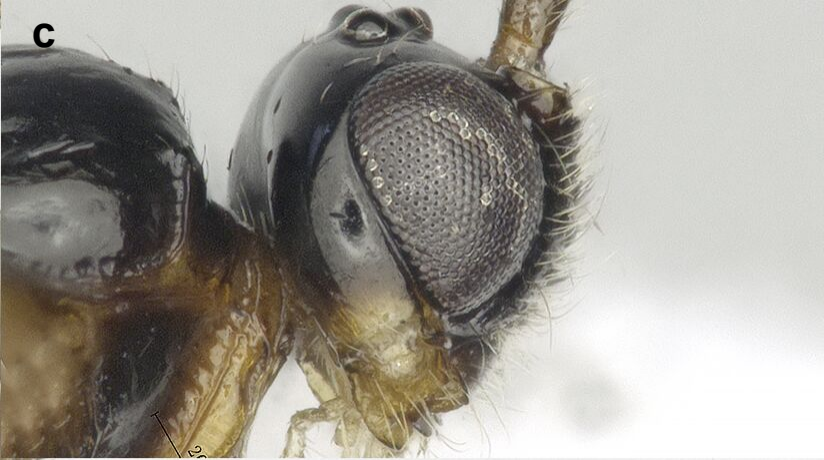
head lateral aspect;

**Figure 3d.**
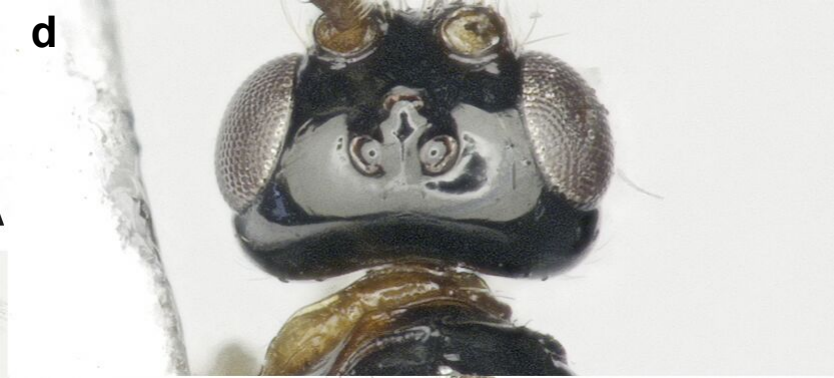
head, dorsal aspect;

**Figure 3e.**
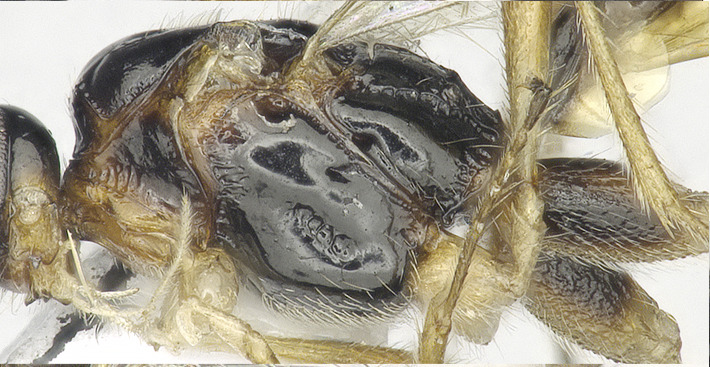
mesosoma, lateral aspect;

**Figure 3f.**
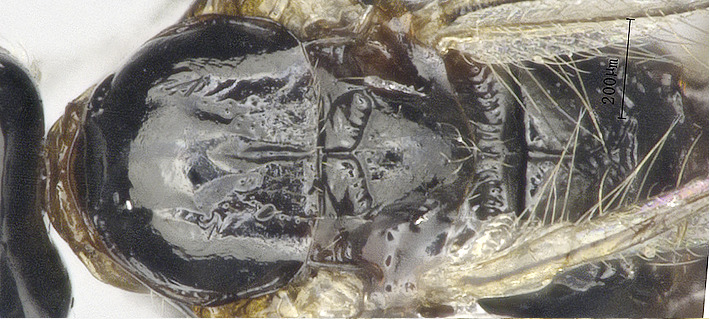
mesosoma, dorsal aspect.

**Figure 4a.**
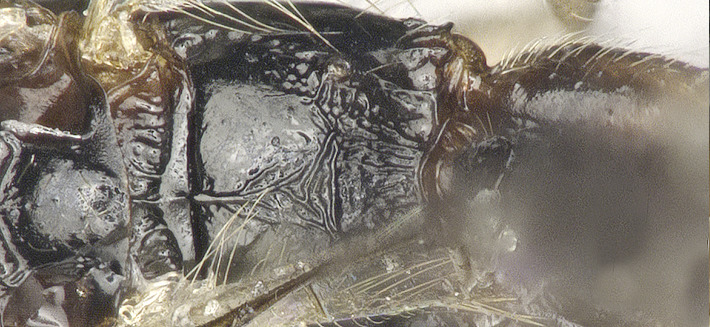
propodeum, dorsal aspect;

**Figure 4b.**
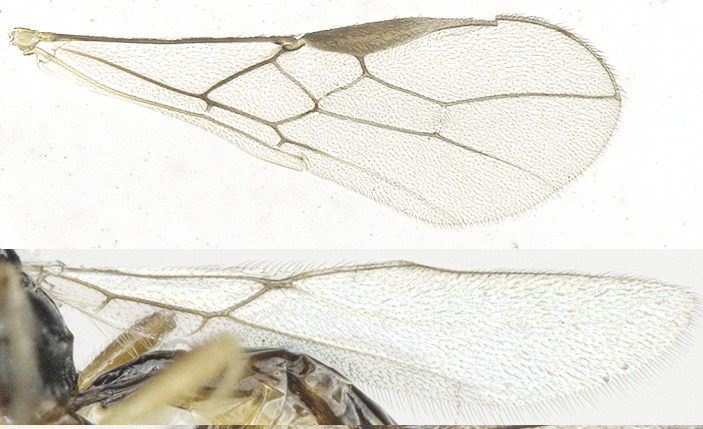
wings;

**Figure 4c.**
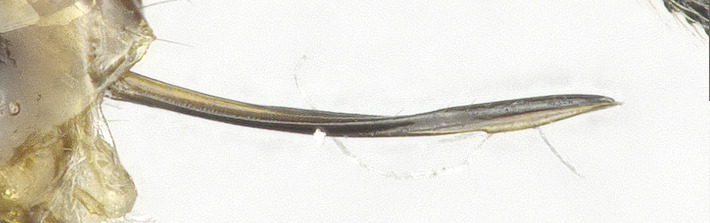
apex of ovipositor, lateral aspect;

**Figure 4d.**
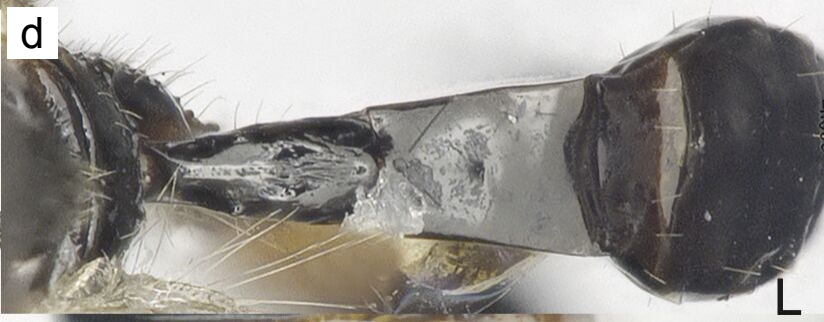
first metasomal tergite, dorsal aspect;

**Figure 4e.**
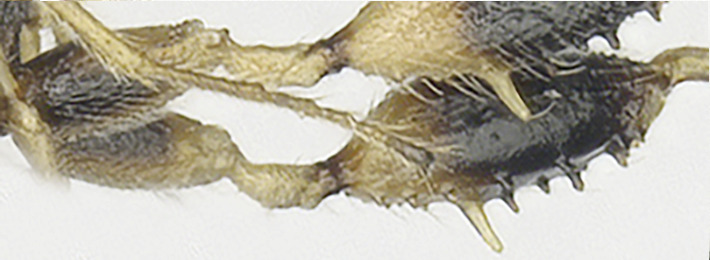


**Figure 4f.**
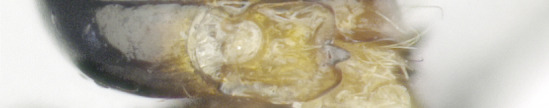
mandible, full view on third tooth.

## References

[B8358175] Belokobylskij S. A., Kostromina T. S. (2011). Two late-spring braconid genera of the subfamily Alysiinae (Hymenoptera: Braconidae) new for the fauna of Russia. Zoosystematica Rossica.

[B8358184] Bhat S. (1980). A new genus of Alysiini (Hymenoptera: Braconidae: Alysiinae). Entomon.

[B8358193] Chen Xue Xin, van Achterberg C. (2019). Systematics, phylogeny and evolution of braconid wasps: 30 years of progress. Annual Review of Entomology.

[B8358202] Gupta A., van Achterberg C. (2022). Review of the Indian species of the genus *Eurymeros* Bhat (Braconidae: Alysiinae) with some nomenclatural changes. Zootaxa.

[B8358211] Jasso-Martínez J M, Santos B F, Zaldívar-Riverón A, Fernandez-Triana J, Sharanowski B J, Richter R (2022). Phylogenomics of braconid wasps (Hymenoptera, Braconidae) sheds light on classification and the evolution of parasitoid life history traits. Molecular Phylogenetics and Evolution.

[B8358224] Sharma V. (1983). On genus *Euremeros* Bhat with description of two new species (Hymenoptera, Braconidae, Alysiinae). Reichenbachia.

[B8358233] Shaw M. R., Huddleston T. (1991). Classification and biology of braconid wasps (Hymenoptera: Braconidae). Handbooks for identification of British Insects.

[B8364849] van Achterberg C. (1988). Revision of the subfamily Blacinae Foerster (Hymenoptera, Braconidae). Zoologische Verhandelingen Leiden.

[B8358251] van Achterberg C. (1990). Illustrated key to the subfamilies of the Holarctic Braconidae (Hymenoptera: Ichneumonoidea). Zoologische Mededelingen Leiden.

[B8358260] van Achterberg C. (1993). Illustrated key to the subfamilies of the Braconidae (Hymenoptera: Ichneumonoidea). Zoologische Verhandelingen Leiden.

[B8358269] Wharton R. A. (1984). Biology of the Alysiini (Hymenoptera: Braconidae), parasitoids of cyclorrhaphous Diptera. Texas Agricultural Experimental Station. Technical Monograph.

[B8442661] Yu D. S., van Achterberg C., Horstmann K. Taxapad 2016, Ichneumonoidea 2015.. www.taxapad.com.

[B8358287] Zhu J. C., van Achterberg C., Chen X. X. (2017). An illustrated key to the genera and subgenera of the Alysiini from China (Hymenoptera, Braconidae, Alysiinae). ZooKeys.

